# Hyperspectral placenta dataset: Hyperspectral image acquisition, annotations, and processing of biological tissues in microsurgical training

**DOI:** 10.1016/j.dib.2023.109526

**Published:** 2023-08-28

**Authors:** Sami Puustinen, Joni Hyttinen, Antti-Pekka Elomaa, Hana Vrzáková

**Affiliations:** a Microsurgery Center of Eastern Finland, Kuopio University Hospital, Puijonlaaksontie 2, Kuopio 70210, Finland; b Faculty of Science, Forestry and Technology, University of Eastern Finland, Yliopistokatu 2, Joensuu 80100, Finland; c Department of Neurosurgery, Kuopio University Hospital, Puijonlaaksontie 2, Kuopio 70210, Finland

**Keywords:** Medical hyperspectral imaging, Microsurgical training, Tissue classification

## Abstract

The dataset consists of 101 hyperspectral images of four human placentas and six hyperspectral images of contrast dyes (i.e., indocyanine green and red and blue food colorant) that were captured in the range 515–900 nm, step = 5 nm. The hyperspectral images were manually annotated, delineating the key anatomical structures: arteries, veins, stroma, and the umbilical cord. Standard reference materials were used for flat-field correction. The dataset is instrumental for advancing machine-learning algorithms and automated classification of anatomical structures, particularly the classification of superficial and deep vessels and transparent tissue layers.

**Specifications Table**

**Table:** 

Subject	Medical research data
Specific subject area	Medical hyperspectral imaging of human placenta tissues
Type of data	Images (TIFF: Tag Image File Format) Annotations (CSV-like format) Table specifying the images, imaging parameters and annotated tissues Python and MATLAB code and code examples for reading the data
How the data were acquired	The images were acquired using a hyperspectral camera (Senop HSC-2, Senop Oy) mounted to an operating microscope (OPMI Pentero 900, Carl Zeiss AG). The microscope's light source (2×300W Xenon bulbs) was used during the hyperspectral image acquisition. Prior to the image acquisition, the HSI system was calibrated using the reference materials (white and dark reference). The images were acquired, and the relevant parameters, including exposure times, imaging distances, illumination conditions, and focus, zoom, and light intensity settings of the operating microscope were documented. The acquired hyperspectral images were flat-field corrected using a standard white and dark-current references. Key anatomical structures and specular reflections in the hyperspectral images were annotated using a custom-made software (SpimLab 09/2021).
Data format	Raw Analyzed
Description of data collection	Data was collected in the wet lab at Microsurgery Center of Eastern Finland, Kuopio University Hospital. The wetlab was protected with separating curtains that minimized external ambient illumination. Placenta tissues were refrigerated for 24–48 h in a medical refrigerator (2°C) and warmed to room temperature prior to the data collection. Placenta's main vessels were injected with three colorants (i.e., red and blue food dye, indocyanine green).
Data source location	Kuopio University Hospital, Microsurgery Center of Eastern Finland Kuopio, Finland
Data accessibility	Repository name: Zenodo Data identification number: 10.5281/zenodo.8045940 Direct URL to data: 10.5281/zenodo.8045940 The dataset is made available under “Creative Commons Attribution-NonCommercial-ShareAlike 4.0 International Public License”-license. The SpimLab annotation software is available at 10.5281/zenodo.8046363.

## 1 Value of the Data

• The dataset is the only hyperspectral image dataset that combines factors of authentic microsurgical training model, clinical settings and relevance, detailed hyperspectral images, and expert labels.
• The dataset comprises flat field-corrected hyperspectral images and manual labels of key anatomical structures (i.e., umbilical cord, arteries, and veins) in the perfused placenta tissue.
• The dataset was captured under authentic clinical conditions of microsurgical training, i.e., high magnification, surgical illumination, specular reflections, different types of vessels, transparent tissue layers, that contribute to high ecological validity.
• Computer scientists and clinicians will benefit from the dataset by i) better understanding of the spectral properties and the microstructure of the placenta, ii) detecting anomalies that may not be visible to the naked eye, and iii) developing and testing new machine learning algorithms for automated classification of biological tissues, particularly the classification of superficial and deep vessels and transparent tissue layers (similar to subarachnoid spaces).

## 2 Objective

Surgical applications of hyperspectral imaging (HSI) have experienced significant growth over the past decade [1]. One of the primary applications involves utilizing machine-learning techniques to analyze hyperspectral data and improve intraoperative tissue classification. This task can be particularly challenging in microsurgery [2]. However, there is currently a scarcity of clinical hyperspectral data available for training classification algorithms. Moreover, each application requires a specific dataset, and no such dataset exists for microsurgical training or human placentas.

The human placenta is a well-established simulation model in microsurgical training, allowing authentic tissue handling in important microsurgical tasks, such as blunt dissection and anastomosis [3,4]. The placenta comprises various structures, such as superficial and deep blood vessels, membranes, and stroma. Due to the diverse vasculature, the placenta is particularly feasible for training classification algorithms for blood vessel segmentation, which is a central task in intraoperative HSI applications [2,5,6]. Different contrast dyes commonly used in microsurgical training (red and blue food colorant) or clinical practice (indocyanine green, ICG) were incorporated into the dataset to differentiate between the placental arteries and veins, a task that is challenging for the naked eye. The red dye is used to highlight the surface arteries, and the blue dye highlights the surface veins. As a near-infrared dye, ICG is visualized in wavelengths >800 nm and may help to visualize the subsurface arteries. The colorants also allow easier detection of vessel perforations, which are cued by dye leakage.

## 3 Data Description

Flat-field corrected hyperspectral images, SpimLab-annotation files, and bitmap segmentation masks generated from the annotation files are provided in dedicated zip files. The zip-files containing placenta images follow naming scheme “Placenta Pxxx-Pyyy zzz.zip”, where xxx and yyy denote the range of images included in this zip-file, and zzz names the dyes used during imaging. The xxx and yyy are unique numeric identifiers and do not carry semantic meaning. For example, zip-file “Placenta P001-P006 no dye.zip” contains placenta spectral images from the imaging session where no dyes were used during imaging. Further, the zip-file contains files “P001.tif”, “P001.csv”, and “P001, masks.tif”. The first file is the hyperspectral image in TIFF-image format, the second file contains the SpimLab-annotations, and the third file contains a bitmap mask render of the SpimLab-annotations in TIFF-image format. We also provide hyperspectral images of contrast dye samples in “Dye samples.zip”, following the same convention as placenta zip-files. The dataset contains 101 flat-field corrected hyperspectral images of human placentas and six images of contrast dyes. The flat-field corrected data could be reconstructed from the provided raw measurement data. The ENVI-formatted spectral images of human placenta, dye sample, and reference raw measurement data are provided in “Raw measurement data.zip”. Preprocessed white and dark-current references, white reference spectrum, and a definition file describing the preprocessing and flat-field correction procedures are provided in “References.zip”.

Since the dataset contains data in several formats, helper functions for reading the data have been provided in file “Code.zip”. A Markdown document “Usage notes.md” contains examples for reading the spectral images and mask files. Other files in the archive are “read_envi.py” containing Python functions for reading data in ENVI-format, “spectral_tiffs.py” containing Python functions for reading and writing spectral image TIFF files and mask TIFF files, and “annotations.py” containing functions for rendering annotations created in SpimLab into bitmap raster mask images. Table 1 illustrates the structure of the dataset.

**Table 1:** Structure of the published dataset.

Files	Description	Number of files	Format
Placenta_HSI_description.xlsx	Imaging parameters and annotated tissue classes for each hyperspectral image	1	.xlsx
Code.zip	Helper functions for Python environments and usage note	4	*.md, *.py
Dye samples.zip	Dye samples	18	*.csv, *.tif
Placenta P001 - P006 no dye.zip	Placenta images	18	*.csv, *.tif
Placenta P007 - P030 red blue.zip	Placenta images	72	*.csv, *.tif
Placenta P031 - P053 red blue.zip	Placenta images	69	*.csv, *.tif
Placenta P054 - P077 ICG.zip	Placenta images	72	*.csv, *.tif
Placenta P078 - P101 ICG.zip	Placenta images	72	*.csv, *.tif
Raw measurement data.zip	All raw measurement data	234	ENVI (*.hdr, *.dat)
References.zip	Preprocessed references	14	*.tif, *.json, *.txt
Total		569	

## 4 Experimental Design, Materials and Methods

### 4.1 Placenta Preparation

Four human placentas were obtained with the consent from the donors at the Department of Obstetrics at the Kuopio University Hospital. The placentas were refrigerated for 24–48 h in a medical refrigerator (2°C) and naturally warmed to room temperature prior to the hyperspectral image collection. Medical doctor (SP) cannulated the main vessels of the placenta (the umbilical vein and the umbilical arteries) and irrigated the vasculature with saline. Next, dye solutions were prepared by mixing 1 ml of dye (Colour Red / Colour Blue, Dr. Oetker) and 30 ml of water in separate syringes. 7.5 ml of red dye solution was injected in both umbilical arteries and 15 ml of the blue dye solution in the umbilical vein. Indocyanine green (ICG) was prepared by mixing 1 mm × 1 mm ICG powder (Verdye, Diagnostic green) per 10 ml of water in a cup. 30 ml of this solution was prepared and injected in equal amounts (7.5 ml) into the two umbilical arteries in each placenta prior to acquiring the ICG hyperspectral images.

### 4.2 Hyperspectral Image Acquisition in Wet Lab

Dataset was acquired using a customized HSI system [7]. The system comprised a portable hyperspectral camera (Senop HSC-2, Senop Oy), an operating microscope (OPMI Pentero 900, Carl Zeiss AG), optic adapters, and a computer used to control the imaging. The hyperspectral camera used a snapshot-based technique to capture images in the range of 500–900 nm with a resolution of 1024 × 1024 pixels. Prior to capture, the HSI system was calibrated using the reference materials. White reference image was acquired by imaging a standard white reference target (White balance target, Edmund Optics) and dark reference was acquired by covering the hyperspectral camera lens (Blackout Fabrick, Thorlabs).

Image acquisition took place in a wet lab under two illumination conditions. The ambient illumination was minimized with blackout drapes and turning off the ceiling lights (“dim room”) or the images were captured in the microsurgical training illumination under the T5/840 24 W LED ceiling lights (“lit room”). The operating microscope's light source (2×300W xenon) was used with varying light intensities: 30, 42, or 50%. The images were acquired with the microscope's objective lens directly above the sample in angle of ≈ 0 degrees (beampath-placenta surface normal). The shared table “placenta_HSI_description.xlsx” describes all imaging parameters, including exposure times, imaging distances, illumination conditions, and focus, zoom, and light intensity settings of the operating microscope. The parameters of hyperspectral image acquisition are described in Table 2 and a transposed excerpt of the “placenta_HSI_description.xlsx” exemplifies the documented data for a single image P039 (Table 3).

**Table 2:** The parameters of hyperspectral image acquisition.

N	Image acquisition parameters	Value Range
1	Laboratory illumination	Dim or Lit
2	Wavelength Range	515–700 nm; 515–900 nm
3	Number of bands	38–78
4	Wavelength increment	5 nm
5	Exposure time	175 ms
6	Microscope light intensity	30–50% (Zeiss OPMI Pentero 900)
7	Microscope magnification	2x–6.3x
8	Microscope focus	197–343 mm
9	Microscope mode	RGB or IR800
10	Imaging distance	330–480 mm
11	Angle between beampath – placenta surface normal	≈ 0 degrees
12	Angle between optical path - illumination	≈ 0 degrees
13	Injected dye	Blue dye, Red dye, ICG dye, Residual blue dye in veins, Residual red dye in arteries

**Table 3:** A transposed excerpt of placenta_HSI_description.xlsx showing the structure of the sheet.

Data	HSI Image File	P039.tif
	Annotation File	P039.csv
	Segmentation Bitmap File	P039, masks.tif
	Archive File	Placenta P031 - P053 red blue.zip
	Type	Placenta
	Dye	red, blue
Imaging parameters	Wetlab illumination (dim/lit)	lit
	Range (nm)	515–700
	Bands	38
	Light intensity (%)	30
	Magnification (x)	2
	Focus (mm)	343
	Imaging distance (cm)	33
Tissue classes [pixels]	Total segmented	837395
	Artery	172585
	Artery, ICG	
	Blue dye	
	ICG	
	Red dye	
	Stroma	467245
	Stroma, ICG	
	Suture	
	Umbilical cord	88043
	Vein	109522
	Segmented by	Students

### 4.3 Preprocessing and Corrections of Hyperspectral Images

Spectral cropping was applied to limit the data range to 515–700 nm. Due to technical issues in the spectral camera or elsewhere in the imaging setup, the first bands at 500–510 nm were misaligned and omitted from the analysis. Reflectance spectral images S(x,y,λ) were computed from the raw spectral images $s_{i}\left(x,y,\lambda \right)\;$with the flat-field correction:

$$S\left(x,y,\lambda \right)=\frac{s_{i}\left(x,y,\lambda \right)-s_{d}\left(x,y,\lambda \right)}{s_{w}\left(x,y,\lambda \right)-s_{d}\left(x,y,\lambda \right)}\times R_{w}\left(\lambda \right).$$

where $s_{d}\left(x,y,\lambda \right)$ is the raw spectral image of dark-current reference, $s_{w}\left(x,y,\lambda \right)\;$is the raw spectral image of the white reference, and $R_{w}\left(\lambda \right)\;$is the known reflectance of $s_{w}\left(x,y,\lambda \right)$ [8]. In the ideal case, Eq. (1) is bounded in zero to one range. Specular reflections, low signal, or even fluorescence, however, caused the corrected data to exceed the expected range. The analysis of the errors showed that it was sufficient to clamp the computed reflectance data to zero to one range without losing meaningful information. The spectral camera had another problematic spectral area at 640 nm, where the camera switched sensors. Our Senop HSC-2 model comprised of two separate Fabry-Perot interferometers. At their junction point, approximately at 640±5 nm, a non-contiguous anomaly affected the sensitivity of the data acquisition. The reflection spectra had a significant drop at this range. This was a systematic error in the measured dataset and the users of the dataset should account for this in their studies.

The flat-field corrected reflectance spectral images were saved as 16-bit multipage TIFF-images. The first page of each TIFF-image is an RGB-render of the spectral image, followed by individual band images in the subsequent pages as in [9]. The RGB renders have been computed by first converting the reflectance spectral image into a CIE XYZ coordinate system [10] by assuming the imaged scene is illuminated by the CIE D65 light source [10]. CIE 1931 color-matching functions were used. For a reflective case [10], the conversion functions are:

$$\begin{array}{c} X\left(p\right)=\frac{100}{N}\int_{\lambda }S\left(\lambda ,\;p\right)I\left(\lambda \right)\bar{x}\left(\lambda \right)d\lambda \\ \begin{array}{c} Y\left(p\right)=\frac{100}{N}\int_{\lambda }S\left(\lambda ,p\right)I\left(\lambda \right)\bar{y}\left(\lambda \right)d\lambda ,\\ Z\left(p\right)=\frac{100}{N}\int_{\lambda }S\left(\lambda ,p\right)I\left(\lambda \right)\bar{z}\left(\lambda \right)d\lambda \end{array} \end{array}$$

where S(p,λ) is the reflectance at a spatial spectral image coordinate p, I(λ) is the spectrum of the CIE D65 illuminant, and $\bar{x}\left(\lambda \right)$, $\bar{y}\left(\lambda \right)$, and $\bar{z}\left(\lambda \right)$ are the color matching functions, and N is the normalization term:

$$N=\int_{\lambda }I\left(\lambda \right)\bar{y}\left(\lambda \right)d\lambda .$$

The CIE XYZ image was then converted into an RGB coordinate system [10] by matrix multiplication

$$\left[\begin{array}{c} R\left(p\right)\\ G\left(p\right)\\ B\left(p\right) \end{array}\right]=\left[\begin{array}{ccc} 3.2406 & -1.5372 & -0.4986\\ -0.9689 & 1.8758 & 0.0415\\ 0.0557 & -0.2040 & 1.0570 \end{array}\right]\left[\begin{array}{c} X\left(p\right)\\ Y\left(p\right)\\ Z\left(p\right) \end{array}\right].$$

Each coordinate C(p) of R(p), B(p), and G(p) was scaled and clipped into 0≤C(p)≤1 range, followed by gamma-correction with equation:

$$\gamma \left(C\left(p\right)\right)=\left\{ \begin{array}{c} 12.92C\left(p\right),\;C\left(p\right)\leq 0.0031308\\ 1.055C{\left(p\right)}^{\frac{1}{2.4}}-0.055,\;\text{otherwise}. \end{array}\right.$$

Finally, the resulting coordinates were scaled into 8-bit unsigned integers and the image was saved as the first page of the multi-page spectral image TIFF-image.

The colorimetric functions were defined for the range 360–830 nm. The spectral imaging ranges covered two ranges 515–700 nm and 515–900 nm. Since the color image renders were not color accurate representations of the original samples, the placenta samples tended to look yellowish.

### 4.4 Hyperspectral Image Annotations

**Fig. 1:**
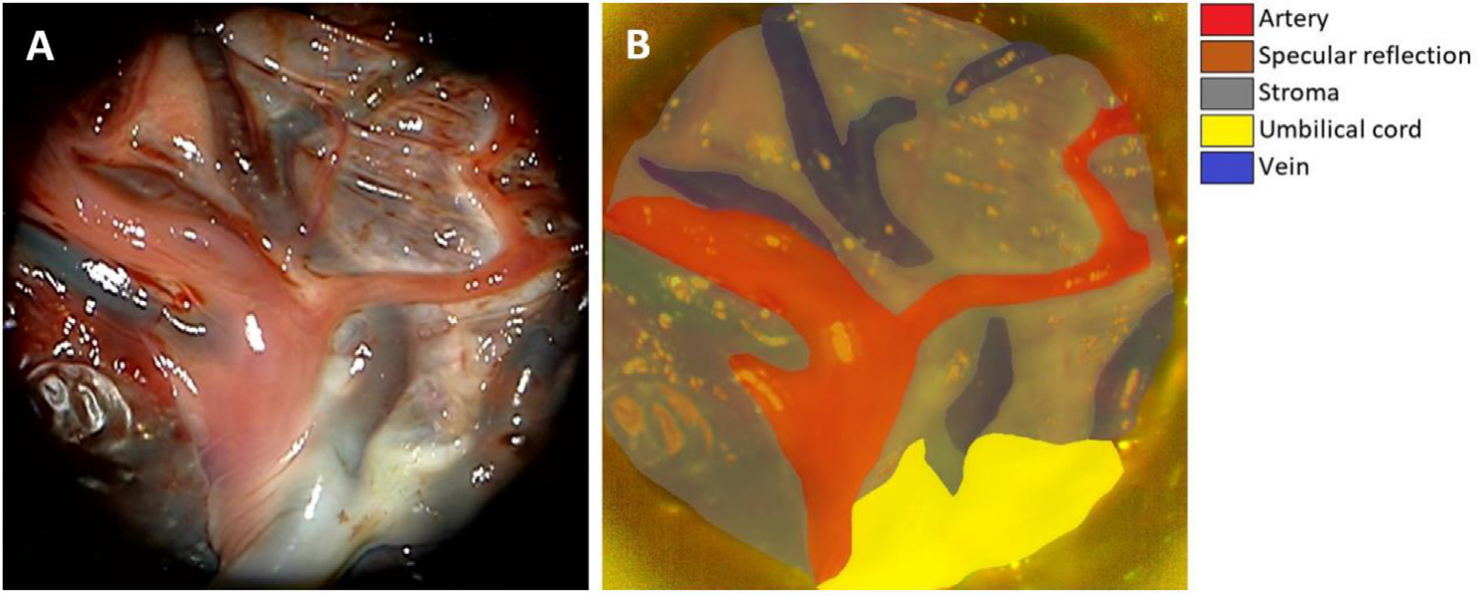
Illustration of the human placenta and the annotated tissues. Operating microscope view of the human placenta tissues near the umbilical cord after the injection of contrast dyes (A) and corresponding hyperspectral image P039 color render with the annotations (B).

Six hyperspectral images with the contrast dye and the tissue anatomy (blood vessels, stroma, umbilical cord), surgical sutures and specular reflections of 75 hyperspectral images were annotated by a medical doctor (SP). The remaining 26 images were annotated by University of Eastern Finland computer science master program students in groups. The placenta anatomy and annotation process were taught to the students by SP. The final annotations were evaluated and corrected for anatomical accuracy by SP. In total the annotation process took approximately 40 h (25 h for SP and 15 for students). The annotations are provided both in the CSV-like format and in bitmap format as described in [9]. Fig. 1 represents an example image from the dataset (P039), illustrating the anatomy and annotations of the placenta tissue.

## Ethics Statements

This study was performed in line with the principles of the Declaration of Helsinki and the ethical use of human material. The placentas were collected with consent from the donors at the Kuopio University Hospital Department of Obstetrics. The approval for the study was granted by the Hospital District of Northern Savo Ethics Committee (PSSHP TET 144/2017).

## CRediT authorship contribution statement

**Sami Puustinen:** Conceptualization, Data curation, Formal analysis, Investigation, Methodology, Validation, Visualization, Writing – original draft, Writing – review & editing. **Joni Hyttinen:** Formal analysis, Methodology, Software, Visualization, Writing – original draft, Writing – review & editing. **Antti-Pekka Elomaa:** Funding acquisition, Project administration, Resources, Validation, Writing – original draft, Writing – review & editing. **Hana Vrzáková:** Conceptualization, Formal analysis, Investigation, Methodology, Writing – original draft, Writing – review & editing.

## Data Availability

Hyperspectral Placenta Dataset: Hyperspectral Image Acquisition, Annotations, and Processing of Biological Tissues in Microsurgical Training (Original data) (Zenodo).
